# Pros and cons of streamlining and use of computerised clinical decision support systems to future-proof oncological multidisciplinary team meetings

**DOI:** 10.3389/fonc.2023.1178165

**Published:** 2023-05-18

**Authors:** Janneke E. W. Walraven, Rob H. A. Verhoeven, Jacobus J. M. van der Hoeven, Renske van der Meulen, Valery E. P. P. Lemmens, Gijs Hesselink, Ingrid M. E. Desar

**Affiliations:** ^1^ Department of Medical Oncology, Radboud University Medical Center, Nijmegen, Netherlands; ^2^ Department of Research, Netherlands Comprehensive Cancer Organization (IKNL), Utrecht, Netherlands; ^3^ Department of Medical Oncology, Amsterdam University Medical Center location University of Amsterdam, Amsterdam, Netherlands; ^4^ Department of Intensive Care, Radboud Institute for Health Sciences, Radboud University Medical Center, Nijmegen, Netherlands

**Keywords:** multidisciplinary team meeting, oncology, streamlining, computerized clinical decision support systems, oncology care

## Abstract

**Introduction:**

Nowadays nearly every patient with cancer is discussed in a multidisciplinary team meeting (MDTM) to determine an optimal treatment plan. The growth in the number of patients to be discussed is unsustainable. Streamlining and use of computerised clinical decision support systems (CCDSSs) are two major ways to restructure MDTMs. Streamlining is the process of selecting the patients who need to be discussed and in which type of MDTM. Using CCDSSs, patient data is automatically loaded into the minutes and a guideline-based treatment proposal is generated. We aimed to identify the pros and cons of streamlining and CCDSSs.

**Methods:**

Semi-structured interviews were conducted with Dutch MDTM participants. With purposive sampling we maximised variation in participants’ characteristics. Interview data were thematically analysed.

**Results:**

Thirty-five interviews were analysed. All interviewees agreed on the need to change the current MDTM workflow. Streamlining suggestions were thematised based on standard and complex cases and the location of the MDTM (i.e. local, regional or nationwide). Interviewees suggested easing the pressure on MDTMs by discussing standard cases briefly, not at all, or outside the MDTM with only two to three specialists. Complex cases should be discussed in tumour-type-specific regional MDTMs and highly complex cases by regional/nationwide expert teams. Categorizing patients as standard or complex was found to be the greatest challenge of streamlining. CCDSSs were recognised as promising, although none of the interviewees had made use of them. The assumed advantage was their capacity to generate protocolised treatment proposals based on automatically uploaded patient data, to unify treatment proposals and to facilitate research. However, they were thought to limit the freedom to deviate from the treatment advice.

**Conclusion:**

To make oncological MDTMs sustainable, methods of streamlining should be developed and introduced. Physicians still have doubts about the value of CCDSSs.

## Introduction

To provide high-quality, comprehensive and holistic oncological care, it has become common practice to discuss nearly all patients with cancer at least once in oncological multidisciplinary team meetings (MDTMs) (1). In these (usually weekly) meetings lasting one to two hours, outcomes of diagnostics and patient and disease characteristics are discussed, with the intention of determining an optimal multidisciplinary treatment plan (2). MDTMs are attended by medical specialists, and in university/training hospitals also by residents (defined as graduate doctors, in training to become medical specialists), from all involved specialties, including medical, radiation and surgical oncology, pathology, radiology, and nuclear radiology. In addition, MDTMs are often attended by clinical nurse specialists and have administrative support (3).

Discussing a patient in the MDTM is an important step in the treatment process, since it improves tumour staging, decision-making, and possibly survival (4–6). In addition, MDTMs can also have educational aspects and are used for quality assurance (7, 8). Several national guidelines therefore require that every patient with cancer should be discussed at least once in a MDTM (9–12). Not infrequently, however, a patient is discussed several times: preoperatively to discuss diagnosis and treatment plan, before and after neo-adjuvant treatment, postoperatively to determine the Tumour-Node-Metastasis (TNM) stage, additional therapies and follow-up plan (13). Patients may also be discussed in the setting of recurrent disease and in the palliative phase (9). New complex treatment options, multimodality treatments and more aggressive approaches to oligometastatic disease all increase the need to discuss patients in MDTMs more frequently than before (14). Moreover, as cancer incidence continues to rise, the number of new patients to be discussed is also increasing (15).

In most western countries, there are different types of oncological MDTMs (e.g. local MDTMs, regional MDTMs, regional/nationwide expert teams). Local MDTMs are attended by medical specialists from one (non-teaching) peripheral hospital, sometimes with the participation of an academic or regional expert specialist as an advisor. At the beginning of this century there were many general MDTMs (where different tumour-types are discussed within one MDTM), but nowadays the majority of them have been replaced by tumour-type-specific MDTMs. Many hospitals have established interhospital collaborations, which has led to regional networks of collaborating hospitals who jointly participate in one tumour-type-specific MDTM. Communication mainly takes place via videoconferencing, sometimes via audioconferencing. The composition of regional MDTMs varies, although a regional or academic expert hospital is always affiliated. Developing regional MDTM networks is common practice in many countries worldwide and this process has only accelerated in the COVID-19 era (16–18). Regional/nationwide expert teams consist of a number of super-specialised specialists from across the country or broader region, brought together via videoconference, who are consulted if the local/regional MDTM does not reach consensus. This can be on a regular basis (i.e. every two to four weeks) or on demand. Patients are often discussed in several MDTMs (8).

A number of studies investigated the amount of discussion time per case and found different results, with a median discussion time per case ranging from two to four minutes (19–22). Shorter discussion time results in decreased sharing of patient-centred information (e.g. psychosocial information and patient’s view) (22). Lack of patient-centeredness leads to greater inability to reach decisions (23, 24). The decision-making process is also found to be negatively affected by prolonged MDTMs and time/workload pressure (25–27). In addition, time pressure forces MDTMs into a more business-like atmosphere, which harms team dynamics between participants (23, 28). Previously, we reported facilitators and barriers on high quality and well-functioning MDTMs (29).

There is much concern about how MDTMs currently work. The perceived time pressure is not yet at the expense of good quality patient care (30–32). Therefore, there is an urgent need to reduce the time commitment for participants in MDTMs to continue providing optimal care for patients with cancer in the near future. After an extensive and systematic literature search we identified two major ways to reduce the time constraints on oncological MDTMs: streamlining and using computerised clinical decision support systems (CCDSSs) (33). Streamlining is the process of selecting the patients who need to be discussed and in which type of MDTM (8). CCDSSs are interactive software systems that are designed to help clinicians with decision-making tasks, such as determining a diagnosis based on automatically uploaded patient data into the minutes, or recommending for a guideline-based treatment proposal for the patient (34, 35). These CCDSSs aim to reduce unjustified practice variations and improve the quality of care (36). For example, the use of OncoDoc2, a CCDSS implemented in breast cancer MDTMs in France, was found to improve the decision compliance rate with the reference guidelines from 73% to 93% after its implementation (37).

In this study we aim to identify the pros and cons of a number of suggestions for streamlining and use of CCDSSs, as perceived by medical specialists and residents participating in MDTMs on a regular basis. Our results will further shape the debate on ways to restructure and future-proof MDTMs.

## Methods

### Study design

Between May 2018 and May 2019 a qualitative semi-structured interview study was conducted among participants in oncological MDTMs. We followed the COREQ (consolidated criteria for reporting qualitative research) checklist (**Supplement A**). The study was approved by the local ethics committee (CMO Arnhem – Nijmegen: registration number ECSW-L T-2022-5-11-24356). All interviewees agreed to participate after reading written information about the project and its aims, and their consent was formally recorded.

### Interviewees

Interviewees participated in oncological MDTMs on a regular (e.g. weekly) basis. In order to maximise variation in interviewees’ professional and demographic characteristics, we purposively sampled (38) interviewees based on five criteria: 1) sex; 2) medical specialist versus residents; 3) type of hospital (peripheral or academic medical centre) 4) region of hospital (coded to A-B-C-D, based on the provinces in the Netherlands) and 5) specialty (surgical, medical and radiation oncology, radiology, nuclear radiology and pathology). Interviewees were approached by email by two researchers (ID and JW) to participate in our study.

### Data collection

The primary researcher (JW) conducted semi-structured interviews. JW is a medical oncologist who has been attending two MDTMs per week for over five years and received interview training prior to the study from an experienced researcher in the field of qualitative research (GH). Interviews were conducted using a topic guide, which was evaluated and adjusted if necessary after each interview. The main topics that guided question development were: the current MDTM setting, MDTM workload and ideas on future-proofing MDTMs with emphasis on streamlining and CCDSSs (**Supplement B**).

During the interviews JW used probes, took notes and summarised statements to fully comprehend and validate interviewees’ perspectives. All interviewees gave their consent prior to each interview and were given the opportunity to comment and reflect on the accuracy and validity of the information obtained. All interviews were audiotaped and transcribed verbatim. Interviews had a median duration of 38.7 minutes and lasted between 27 and 72 minutes. It is worth noting that the interviews were also conducted to gain insight into the current perceived quality of MDTMs and whether they serve educational purposes for residents (29, 39). These findings are beyond the scope of this article. The transcripts were loaded and stored on secure servers at the hospital where the researchers work, using ATLAS.ti software version 8.0, a software program for detailed coding in qualitative data analysis.

### Data analysis

The data was analysed using thematic analysis, with the unit of analysis being the recorded interview. In thematic analysis researchers familiarise themselves with the data through a process of reading and re-reading, generating initial codes, finding overarching themes and revising those themes (40). Three researchers (JW, AO, RM) were involved in reviewing and analysing the interview transcripts. AO and RM had different backgrounds from JW to ensure different reflexive positions (AO is a health scientist, RM is a student of biomedicine). Relevant data was identified and structured using open, axial and selective coding. Coding is the interpretive process by which conceptual labels are given to the data (41). Initially, all three researchers independently read the transcripts and coded relevant fragments (related to identifying the pros and cons of various suggestions for streamlining and using CCDSSs) to minimise the subjectivity of findings (open coding). After each interview, the transcript was coded before the next interview took place. During the iterative analysis process, researchers regularly shared and discussed the uniqueness and meaning of generated open codes. After discussion, codes were reformulated and those with the same meaning were grouped into one unique code (axial coding). After the open and axial coding of the first 15 interviews, all three researchers reached consensus on a list of codes (codebook) that guided the further coding of the rest of the interviews performed by one researcher (RM). New codes and related text fragments were then discussed with at least one of the other researchers. Finally, in the latter transcripts only data that provided additional insights were coded (selective coding). Data saturation was reached after 35 interviews: i.e. new data no longer provided additional insights in relation to the research question (42). During the iterative analysis process, researchers regularly shared and discussed the meaning and uniqueness of generated open codes. Throughout the analysis JW grouped codes belonging to the same concept into categories and finally identified themes from the data in consultation with other research members involved (RV, ID, GH). Data analysis was supported using a qualitative analysis software program (ATLAS.ti version 8.0).

## Results

Thirty-five individual semi-structured telephone interviews were analysed. Interviewees were evenly divided according to sex, medical specialist versus resident and medical specialties. The distribution of the interviewees across the regions was slightly skewed. (**Table 1**). Furthermore, **Table 1** also shows that the main opinion of the interviewees towards streamlining was slightly more positive (n=20) than negative (n=14), and that more female interviewees were in favour of streamlining (n=12 versus 5), while the pro-con distribution among the male interviewees was evenly distributed (n=8 versus 9). There were no differences in the pro-con streamlining distribution in the other subgroups (i.e. medical specialty, type of hospital, region, resident versus medical specialist, years of experience as specialist, or years in training as resident).

**Table 1:** Characteristics of participants and primary opinion towards streamlining.

	Medical specialist/Resident (n=16/19)	Streamlining Pro^a^ (n=20)	Streamlining Con^a^ (n=14)	Streamlining No opinion^a^ (n=1)
Sex				
Male	9/8	8	9	–
Female	7/11	12	5	1
Medical Specialty				
Surgical oncology	4/4	5	3	–
Medical oncology	3/2	4	1	–
Radiation oncology	3/3	2	1	–
Pathology	3/4	4	3	–
Radiology	2/3	4	3	1
Nuclear radiology	1/3	2	2	–
Hospital				
Academic	7/16	13	10	–
Peripheral	9/3	7	4	1
Region^b^				
A	3/1	3	1	–
B	7/7	9	4	1
C	2/2	2	2	–
D	4/9	6	7	–
Resident versus medical specialist				
Medical specialist	16	10	6	–
Resident	19	10	8	1
Experience as medical specialist (years)				
<5	3	1	2	–
5-10	5	5	–	–
>10	8	4	4	–
Training duration of residents (years)				
≤3	4	2	2	–
4-6	15	8	6	1

a Streamlining: discussing a predefined selection of cases rather than discussing every case in the multidisciplinary team meeting. The division into pro/con/no opinion was based on the most prominent opinion of the participant.

b Regions are coded based on the provinces in the Netherlands.

All interviewees noted that attending and preparing MDTMs is very time-consuming and indicated that the current MDTM workflow needs to be changed in the near future to keep the implementation of MDTMs feasible.

The analysis resulted in the emergence of streamlining suggestions that were thematised based on standard and complex cases and the location of the MDTM (i.e. local MDTM, regional MDTM or regional/nationwide expert teams). (**Table 2**) Furthermore, **Table 2** also lists the associated six categories, with pros and cons per category and corresponding quotes.

**Table 2:** Pros and cons of suggestions for streamlining^a^ oncological multidisciplinary team meetings.

Theme	Category	Pros and cons	Representative quotes
Local^b^ or regional MDTM^c^ – standard cases^d^			
	**Discuss standard cases within local panels of 2-3 specialists outside the MDTM**		
		*Pro*	
		• Creates more time for complex cases in MDTM • Less time investment for specialists not present in local panel	*[Specialist P2]*: “By discussing low-complex – high-volume tumour-type cases within a local panel outside the MDTM, you save time for substantive discussion for the more complex cases.”
		*Con*	
		• Lack of input from non-present specialties in local panel • Lack of input on clinical trials in local panel	*[Resident R1]*: “You miss the input of specialists from specialties that are not represented in the local panel. You simply do not know whether you are missing important input, for example about a clinical trial option.”
	**Refer briefly to standard cases during the MDTM without discussion**		
		*Pro*	
		• Taking minutes for electronic health record • Ability to check on unforeseen details • Little time investment	*[Specialist RO2]*: “Start by briefly naming the simple cases that fall within protocol. Then minutes are taken, there is a check that nothing special has been overlooked, and it does not take up too much time in the MDTM.”
		*Con*	
		• No added value to discussing standard case	*[Specialist N1]*: “A case in which the diagnosis and the resulting treatment plan are crystal clear does not need to be briefly mentioned either. That has no added value.”
	**Do not discuss standard cases in MDTMs at all**		
		*Pro*	
		• No added value to discussing standard cases	*[Specialist R3]:* “I don’t think we should discuss a case simply for the sake of discussing it. We only need to discuss complex cases where a decision has to be made.”
		*Con*	
		• Standard cases do not exist	*[Specialist P3]*: “It is important that all cases are discussed in the MDTM. Sometimes I think in advance that a case is really straightforward, but regularly there appears to be a twist that makes the case complex.”
		• MDTM discussion is an obligation to the patient	*[Specialist S1]*: “Patients will not label themselves as ‘straightforward cases’. It is our duty to the patient to establish a multidisciplinary treatment plan.”
Regional MDTM			
	**Discuss all cases in regional MDTMs, abolish local MDTMs**		
		*Pro*	
		• Contributes to centralisation/subspecialisation of specialists within tumour types	*[Specialist S1]*: “A regional MDTM also forms a good starting point for regional centralisation agreements. We are beyond the illusion that we should all be able and willing to treat all tumour types.”
		*Con*	
		• More patients to discuss in regional MDTM	*[Specialist RO2]*: “If all patients have to be discussed regionally, you will be involved in all the cases of other hospitals, which makes the workload even greater than it already is.”
		• No proper financial compensation available for discussing patients of another hospital	*[Specialist S2]*: “There is currently no suitable payment system for discussing patients outside your own hospital.”
	**Discuss complex cases**^e^ **in regional MDTM, not in local MDTMs**		
		*Pro*	
		• Merging expertise of specialists • Increases uniformity in discussing innovative/clinical trial treatment options	*[Specialist RO3]*: “As a specialist in a smaller peripheral hospital, I am less highly specialised than a colleague from the academic medical centre. There are so many oncological innovations today that it is difficult to keep up. So I would like to hear the opinion of the super-specialist, especially for complex cases.”
		*Con*	
		• Creates relationships of authority	*[Specialist MO2]*: “Power relations play a negative role in regional MDTMs. Who has the most expertise and the final say in discussions, the (often young) specialist from the academic medical centre or the (often more experienced) specialist from a peripheral hospital who treats more patients?”
		• Local MDTM loses expertise	*[Specialist RO1]*: “If complex cases no longer appear in a local MDTM, the local MDTM loses too much expertise.”
Regional/nationwide expert teams^f^			
	**Discuss highly complex cases with a regional/nationwide expert teams**		
		*Pro*	
		• Availability of highly specialised specialists for consultation	*[Resident N1]*: “For highly complex cases where the MDTM cannot reach a decision, it should be possible to refer the matter to a team of super-specialised regional/national specialists.”
		*Con*	
		• Delay in treatment due to loss of time through consultation of highly specialised specialists	*[Resident S2]*: “You must ensure that cases are not too easily regarded as highly complex, as a result of which treatments are unnecessarily postponed because an expert team still has to be consulted.”

S, surgical oncologist; MO, medical oncologist; RO, radiation oncologist; R, radiologist, N, nuclear radiologist; P, pathologist.

MDT, multidisciplinary team; MDTM, multidisciplinary team meeting; upper-GI, upper gastro-intestinal tract.

a Streamlining: discussing a predefined selection of cases rather than discussing every case in the MDTM.

b Local MDTM: an MDTM with specialists from a (non-teaching) peripheral hospital, where sometimes an expert specialist from an academic medical centre participates as an advisor.

c Regional MDTM: one MDTM to which specialists from various regional hospitals, including an expert or reference centre, connect via videoconference.

d Standard cases: low-complex case involving high-volume tumour type that can be treated within protocol.

e Complex cases: high-complex and/or low-volume tumour type case, both requiring multidisciplinary discussion in order to decide on the individual treatment proposal.

f Regional/nationwide expert teams: teams of super-specialized specialists across the country or a broader region, brought together via videoconference, who are consulted if the local/regional MDTM does not reach consensus.

### Suggestions for streamlining

#### Theme 1: local or regional MDTM – standard cases

A standard case was defined as one involving a low-complex patient with a high-volume tumour type that can be treated according to existing protocols. Three categories were found in this theme: 1) discuss standard cases within local panels of two to three specialists outside the MDTM, 2) refer briefly to standard cases during the MDTM without discussion, 3) do not discuss standard cases in MDTMs at all.

The interviewees had different opinions about whether a standard case can be defined. Opponents indicated that each patient case is unique and therefore complex in its own way. They argued that every patient is entitled to a multidisciplinary discussion. Excluding standard cases from MDT discussion entails a risk of missing important information, which can ultimately lead to the formulation of incorrect treatment proposals. In addition, MDTMs have a role in recognising opportunities for patients to participate in clinical trials.

However, those interviewees in favour of distinguishing standard cases from complex cases agreed that a standard case can be defined. They proposed establishing in advance a set of criteria that a patient must meet in order to be classified as a standard case. Time saved by not discussing the standard cases could be used to discuss complex cases in more depth.

Some interviewees identified a middle ground: discussing a standard case in a local panel of two to three specialists outside the MDTM instead of with the complete MDT. Another suggestion was to introduce the standard case in the MDTM briefly without discussion: the time investment would be low, while facilitating a check for details that have been overlooked and producing MDTM minutes for practical and legal considerations.

#### Theme 2: regional MDTM

Two categories were found in this theme: 1) discuss complex cases in regional MDTMs, not in local MDTMs and 2) discuss all cases in regional MDTMs; abolish local MDTMs.

A complex case was defined as one involving a high-complex patient or a low-volume tumour type, both requiring multidisciplinary discussion in order to decide on the individual treatment proposal. Again, the same disagreement among interviewees was noted regarding the feasibility of distinguishing between standard and complex cases. Opponents were concerned that the local MDTM would lose expertise if complex cases were no longer discussed there. They were afraid of authority issues between participants from different hospitals in regional MDTMs. They argued that the number of patients to be discussed in regional MDTMs would increase, forcing them to participate in the discussion of patients from another hospital rather than their own hospital, fearing inefficiency and an increased workload. Besides, they questioned whether there would be appropriate financial compensation for this increase in the workload.

Those interviewees in favour of centralising complex cases to regional MDTMs believed this would create scope for pooling specialist expertise and increasing uniformity of treatment or clinical trial proposals across hospitals. They applauded the fact that regional MDTMs would contribute to further subspecialisation of specialists from peripheral hospitals within tumour types, arguing that the field of oncology is too extensive for medical professionals to be able to undertake in its full breadth.

#### Theme 3: regional/nationwide expert teams

One category was found in this theme: discuss highly complex cases with a regional/nationwide expert team. Most interviewees agreed that for highly complex cases, where the regional MDTM cannot reach consensus, it should be possible to consult a regional or nationwide expert team. However concerns were raised about time delay when consulting such a super-specialised team of experts. Furthermore, interviewees were unable to indicate how often such an expert team should meet and whether this should apply to every tumour type.

### Including the use of CCDSSs

In **Table 3** the pros and cons with associated quotes for the use of CCDSSs were listed.

**Table 3:** Pros and cons of using computerised clinical decision support systems (CCDSSs) within oncological multidisciplinary team meetings (MDTMs).

Pros and cons	Representative quotes
Pro	
• Convenience of automatically uploaded data • Generating guideline-based treatment advice • Treatment plan is more uniform	*[Specialist S3]:* “By using CCDSSs, all necessary data is automatically uploaded and a guideline-based treatment advice is generated. In this way, treatment plans within different hospitals become more uniform.”
• Less error-prone and clear minutes • Facilitates research	*[Specialist P2]*: “When data is uploaded in the MDTM minutes, you not only create clear minutes, but can also conduct research on performance indicators.”
Con	
• Limits transfer options between hospitals	*[Specialist S3]*: “There are various electronic patient record systems in the Netherlands, which complicates the use of CCDSSs at regional MDTMs.”
• Limits options to deviate from guideline-based treatment advice	*[Specialist MO2]*: “If digitised protocolised treatment advice is generated, it would limit the freedom for clinicians to deviate from that advice.”

Most interviewees were unfamiliar with CCDSSs. Those who had heard of CCDSSs all indicated that they expected them to be an improvement for MDTMs. None of them had actual experience with using CCDSSs. The main assumed benefits mentioned were: these systems would create greater uniformity in treatment proposals (since guideline-based treatment advice is automatically uploaded), facilitate research and result in complete and error-free minutes. However, there were also concerns about limiting the freedom for specialists to deviate from automatically generated advice and about technical problems transferring patient data between hospitals working with different electronic patient record systems.

## Discussion

The current way of discussing oncological patients in MDTMs requires a considerable time investment from its participants and puts them under substantial time pressure. All interviewees agreed that there needs to be a change in the current workflow in order to guarantee the quality of MDTMs in the near future. Through streamlining, the MDTM workload can be reduced. Using CCDSSs could help standardise treatment proposals and facilitate research, but whether it would reduce the MDTM workload is unclear. We report on the pros and cons of streamlining and CCDSSs.

The increasing pressure on MDTMs and concerns regarding their sustainability are not unique to the Netherlands. Several other countries (i.e. the UK, USA, France and Australia) have reported on the need to change the current workflow of MDTMs to ensure they will be future-proof (8, 32, 43–46). In a recent national survey in the UK, the majority (69%) of the 1220 participants agreed that streamlining cases would be beneficial, although 25% of participants were against it (32). Different options for performing streamlining were explored in this survey: discuss patients in smaller teams rather than the full MDT (56% agreed), do not discuss patient cases that fit into protocolised treatment pathways at all (45% agreed), and select cases with a few participants in a pre-MDTM for discussion in the main MDTM (63% agreed) (32). These streamlining options are in line with the suggested options we found in this study. Furthermore, we noted a suggestion to improve selection of patients for local or regional MDTMs, and a suggestion regarding the next step for expert team consultation in highly complex cases. An example of a virtual on-demand expert MDTM is an advisory board for pregnant patients with cancer (47).

The most frequently mentioned objection to streamlining was a concern regarding the difficulty of distinguishing a standard case from a complex case in advance, as a result of which patients might receive incorrect treatment advice. Guidelines on how to perform this categorisation were drawn up by Winters et al. in 2021 (8). They described 11 steps to ensure the quality of the streamlining process, including the requirement that the case selection method be robust, coherent and evidence-based, the role of the chairperson strengthened, and the selection criteria audited regularly and frequently (8). Furthermore, in 2020 Soukup et al. developed and validated the tool ‘Measure of case-Discussion Complexity’ (MeDiC), which defined 27 factors that contribute to the complexity of a patient case. These factors, for example increased size (T3/T4), nodes affected or diagnostic uncertainty, can be scored by using the tool. The higher the score, the more complex the case is defined. As part of the streamlining process it was proposed to use the tool in a pre-MDTM in which the (very) high complex cases could be distinguished from the low/moderate complex cases (48). Both the guidelines by Winters et al. and the MeDiC tool are useful implementing strategies for streamlining.

The usefulness of streamlining needs to be monitored from the moment it is introduced. The emphasis should be on its impact on reducing inter-hospital differences with regard to treatment proposals and participation in clinical trials, the frequency with which patients are discussed, changes in the type of MDTM where a patient is discussed, clinical patient outcomes, effects on tumour-type-specific specialisation among specialists, feasibility of the organisation of regional/nationwide MDTM networks, stakeholder satisfaction and of course the perceived effects on time pressure.

Further future improvements must be sought in expanding the opportunities offered by CCDSSs (49, 50). A systematic review of 148 randomised controlled trials investigating the effects and clinical outcomes of CCDSSs in a variety of clinical settings concluded that CCDSSs are effective in improving health-care processes. However, evidence regarding economic, workload and efficiency outcomes were sparse (51). Another systematic review of the evaluation of CCDSS, by Van de Velde et al. (52), mentioned that clinical decision support (CDS) should be loaded automatically rather than on demand and the advice should be displayed on screens. However, adherence to CDS advice proved limited (52). In our study, interviewees were unfamiliar with CCDSSs and were afraid that automatically generated protocolised treatment proposals would limit the freedom to deviate from that treatment advice. In 2020, Klarenbeek et al. analysed the barriers and facilitators for implementing CCDSS in a lung-cancer MDTM and agreed that exposing discrepancies between formal guidelines and contextualised decisions would make specialists legally more vulnerable to criticism from patients. Furthermore, they reported a potential lack of confidence among specialists in the accuracy of system algorithms (50). Nonetheless, numerous facilitators for using CCDSSs were listed, including a more structured way of working and reduced MDTM preparation time and duration (50). Strikingly, the majority of those interviewed in our study had neither heard of CCDSSs nor had a good idea of how the use of CCDSSs could contribute to improving MDTMs, while as MDTM participants they are expected to further roll out the implementation of CCDSSs. The possibilities for CCDSSs are likely to continue to increase in the coming years. We believe that when attention is paid to informing physicians about the opportunities offered by CCDSSs, they will gradually be incorporated into MDTMs. Good IT support is mandatory.

### Limitations

Our results must be interpreted in light of some limitations. First, we conducted our interview study solely in the Netherlands. However, as previously stated, several other countries also reported on the need to change the MDTM workflow. We believe therefore that our findings are relevant worldwide.

Second, we only interviewed medical specialists and residents, as they actively contribute to the MDTM discussion. However, it would be valuable to also include insights from the clinical nurse specialist, administrator or other participants such as pharmacists or genetic counsellors, with regard to future-proofing MDTMs. Moreover, the current research was focused on tumour-type specific MDTMs. It is unsure whether our findings are applicable for cancer generic molecular tumour board meetings. Further research is needed.

Third, since being in favour or against streamlining was asked in all interviewees, frequencies could be reported on that item. On the other identified pros and cons we could not report frequencies due to the semi-structured interview method of this study.

Fourth, we conducted only telephone interviews and are aware that face-to-face interviews might have created a different dynamic or depth. The primary researcher maintained an open attitude at all times, so we believe that this potential disadvantage was minimalised. Using telephone interviews increased the scope for making appointments and possibly even the willingness of the interviewees to participate, as they have busy schedules.

Lastly, due to the medical background of the interviewer as medical oncologist, it is possible that the direction of interviews or the interpretation of the data was unintentionally steered. However, this potential bias was mitigated by extensive interview training and having multiple researchers from different backgrounds involved to analyse the data.

## Conclusions

The pressure on oncological MDTMs has increased dramatically in recent years due to the large number of patients that needs to be discussed. This will lead to an untenable situation in the near future. One way to address this problem is to restructure MDTMs through streamlining: discuss standard cases only briefly, not at all, or outside the MDTM with a local panel of two to three specialists, while more complex cases are discussed in tumour-type-specific regional MDTMs and highly complex cases with regional/nationwide expert teams. We identified several pros and cons in connection with streamlining. The use of CCDSSs is another way that can help future-proof MDTMs, although physicians have some doubts about whether CCDSSs reduce the MDTM workload. CCDSSs can help standardise treatment proposals and facilitate research. Further studies should focus on the implementation of streamlining and of CCDSSs.

## Data availability statement

The raw data supporting the conclusions of this article will be made available by the authors, without undue reservation.

## Ethics statement

The studies involving human participants were reviewed and approved by the local ethics committee (CMO region Arnhem – Nijmegen) (registration number ECSW-LT-2021-11-19-79410). The patients/participants provided their written informed consent to participate in this study. Written informed consent was obtained from the individual(s) for the publication of any potentially identifiable images or data included in this article.

## Author contributions

JW identified the research question, the topic guide, and the research design which was corrected and checked by ID, RM, GH, JH, RV, and VL. JW received extensive interview training from GH; JW and ID invited interviewees to participate. JW performed semi-structured telephone interviews. JW and RM analysed interview transcripts. The codebook was developed and refined and categories and themes emerged in consultation with ID, GH, and RV. JW wrote the manuscript. All authors reviewed the manuscript. All authors contributed to the article and approved the submitted version.
