# Supplementary material for: Pros and cons of streamlining and use of computerised clinical decision support systems to future-proof oncological multidisciplinary team meetings
Source: Front Oncol. 2023 May 18;13:1178165. doi: 10.3389/fonc.2023.1178165 (PMC10233094; doi:10.3389/fonc.2023.1178165)
Supplement: Supplementary file 2 [file DataSheet_2.docx]

**Supplement B: Topic guide for semi-structured interviews to restructure and future-proof oncological multidisciplinary team meetings (MDTMs).**

1. Introduction
   - Introducing, asking for consent to recording, explaining goals.
   - Verifying specialty, resident or medical specialist, number and type of tumour-specific MDTMs interviewee is participating in, and affiliated hospital.
2. Current MDTM Setting
   - How are the MDTM’s organised in your hospital? Are there local / regional MDTMs or both? Are there only tumour type specific MDTMs or also general MDTMs?
   - Which specialists are attending at the MDTM? Are there experts from reference centres present?
   - Does your MDTM uses audio- or videoconferencing?
   - Is there an option to consult a (number of) superspecialist(s) across the country/ broader region for highly complex cases?
3. MDTM workload
   - How do you experience the current MDTM workload? Do you feel that changes in workload are necessary? Why yes/no?
   - Do you feel that it is important to discuss oncological patients in MDTMs? Why yes / no? Do you feel that every oncological patient should be discussed? Why yes/no?
4. Future-proof MDTMs
   - What is needed to improve MDTMs in the near future?
   - How would you suggest to future proof MDTMs? What is needed? What is your own role in this?
   - What are your thoughts on streamlining? Should it be executed? How should it be executed? What do you consider as benefits for streamlining? What are concerns? Can you give examples?
   - Are you familiar with computerized clinical decision support systems (CCDSSs)? If yes, have you worked with them and what are your experiences? If not, would you like to implement CCDSSs in clinical practice? Why yes/no?
